# The Clinical Implications and Molecular Mechanism of CX3CL1 Expression in Urothelial Bladder Cancer

**DOI:** 10.3389/fonc.2021.752860

**Published:** 2021-10-04

**Authors:** Guangliang Jiang, Hui Wang, Da Huang, Yishuo Wu, Weihong Ding, Qidong Zhou, Qiang Ding, Ning Zhang, Rong Na, Ke Xu

**Affiliations:** ^1^ Department of Urology, Ruijin Hospital, Shanghai Jiao Tong University School of Medicine, Shanghai, China; ^2^ Department of Urology, Huashan Hospital, Fudan University, Shanghai, China; ^3^ Fudan Institute of Urology, Huashan Hospital, Fudan University, Shanghai, China; ^4^ Department of Nuclear Medicine, West China Hospital, Sichuan University, Chengdu, China

**Keywords:** CX3CL1, bladder cancer, survival analysis, ERK, MAPK

## Abstract

**Background:**

CX3CL1 is a chemokine that may play important roles in cancer immune regulation. Its mechanism in bladder cancer (BCa) is poorly understood. The objective of the current study was to evaluate the association between CX3CL1 and BCa and the related biological mechanisms.

**Methods:**

A total of 277 patients with BCa were enrolled in the present study. The association between CX3CL1 expression and disease outcome was evaluated. *In vitro* and *in vivo* experiments were performed using the TCCSUP cell line to investigate the function of CX3CL1 in BCa.

**Results:**

Compared with low expression, high expression of CX3CL1 was significantly associated with poorer progression-free survival (hazard ratio [HR]=2.03, 95% confidence interval [95% CI]: 1.26-3.27, P=0.006), cancer-specific survival (HR=2.16, 95% CI: 1.59-2.93, P<0.001), and overall survival (HR=1.55, 95% CI: 1.08-2.24, P=0.039). Multivariable Cox regression analysis suggested that CX3CL1 was an independent prognostic factor for BCa outcomes. *In vitro* and *in vivo* experiments indicated that high expression of CX3CL1 was significantly associated with cell proliferation (P<0.001) and invasion (P<0.001). Gene expression profiling results showed that after CX3CL1 knockdown, CDH1 was significantly upregulated, while ETS1, RAF1, and EIF4E were significantly downregulated. Pathway enrichment analysis suggested that the ERK/MAPK signaling pathway was significantly inhibited (P<0.001).

**Conclusions:**

CX3CL1 is an independent predictor of a poor prognosis in BCa and can promote the proliferation and invasion of BCa cells.

## Introduction

With an estimated 573,000 new cases and 212,000 deaths in 2020, urinary bladder cancer (BCa) has become one of the most common malignancies worldwide (1). In the United States, BCa accounted for 7% of all malignancy cases and ranked fourth among all types of malignancies in 2020 (2). Chemical components from cigarettes and industrial products are well-known risk factors; however, the etiology of BCa is poorly understood.

Previous evidence has shown that the immune system might play important roles in the development and progression of BCa, involving immune surveillance and immune tolerance. In immunoregulatory pathways, chemokines serve as key factors (3). Tumor immunotherapy represented by PD1/PD-L1 has attracted great attention in recent years. The effect of chemokines on tumors has been proven to produce a two-way regulation pattern: first, chemokines may enhance antitumor immunity by promoting immune cell recruitment to the tumor site (4); second, autonomous secretion of chemokines and receptors from tumor cells may promote tumor growth by inhibiting antitumor immunity (5).

A unique class of chemokines-the CX3C family has been reported playing a critical role in tumor immune regulation, in which CX3CL1 is the only member (6). It contains a unique 29nm long pedicle structure (mucus domain) in N prime/C prime. This structure allows chemokines to extend from epithelial cells to contact flowing white blood cells, and is independent of selectins and integrins (7). In addition, it can also induce intracellular calcium flow and chemotaxis without activating integrins (7). The receptor of CX3CL1, CX3CR1, is a transmembrane domain receptor, which mediates cell adhesion to CX3CL1 without G protein activation (8). Therefore, the unique structure and function of CX3CL1 has attracted the attention of many researchers.

As an important molecule in the chemokine family, C-X3-C Motif Chemokine Ligand 1 (CX3CL1) was found to be significantly associated with the development and progression of multiple cancers (9–13). However, the roles of CX3CL1 and its receptors are still controversial in terms of the biological regulation of tumors. Studies have suggested that CX3CL1 exerts its tumor-suppressive effect by activating immune cells, including natural killer T cells, and inhibiting tumor cell aggregation and adhesion (14, 15). For example, in a study of breast cancer, the high expression of CX3CL1 might recruit immune cells, such as CD8+ T cells and NK cells, to exert anti-tumor activity (16). Another study showed that patients with high expression of CX3CL1 and CX3CL1 receptors would have a significantly better prognosis in liver cancer (15). However, CX3CL1 may also produce a cancer-promotive effect as an adhesion molecule (9, 13). It was reported that high expression of CX3CL1 can induce tumor cell proliferation and promote the transformation of the cell cycle to S phase in gastric cancer (17). Shulby et al. indicated that CX3CL1 involved in the invasion process of prostate cancer metastasis to bone marrow which could be significantly reduced by anti-CX3CL1 antibody (13). This two-way regulation pattern of CX3CL1 is still unclear in BCa.

The objective of the current study was to evaluate the association between CX3CL1 and BCa and to investigate the underlying biological mechanisms involving CX3CL1 and its receptors in BCa.

## Materials and Methods

### Fresh Tissue Sample

Seven fresh tumor tissue samples of bladder cancer and normal bladder mucosa tissue were collected by TURBT (transurethral resection of bladder tumor). In the end, the specimens due to severe burning and too small volume were eliminated. The qualified samples were divided into two groups (4 cases in the normal tissue group; 5 cases in the tumor tissue group) for Real-Time PCR experiment to determine the difference in CX3CL1 mRNA between the two groups.

### Clinical Samples

Patients with BCa undergoing surgical treatment were enrolled in the present study between December 2011 and December 2016 at Huashan Hospital (Shanghai, China). The inclusion criteria were as follows: (a) sufficient clinical information and follow-up information were available, (b) pathological diagnosis and grading were independently confirmed by three pathologists as transitional cell carcinoma of the bladder, and (c) sufficient formalin-fixed paraffin-embedded (FFPE) specimens could be obtained for further investigation. Informed consent was obtained from all patients for institutional biobank, and the study was approved by the Institutional Review Board at Huashan Hospital. Pathological grading and staging were performed according to the WHO/ISUP (2004) and UICC-TNM (2002) guidelines (18, 19). Ta-T1 was defined as non-muscle-invasive BCa (NMIBC), and T2-T4 were defined as muscle-invasive BCa (MIBC).

### Immunohistochemistry

Immunohistochemistry staining of FFPE samples was performed using an anti-CX3CL1 antibody (Abcam, Cambridge, UK; 1:100) and the avidin-biotin-complex method. The extent and intensity of tumor regions (without necrotic tissue) were further evaluated under a Nikon 80i microscope (Tokyo, Japan). Staining intensity was categorized as 0 (absent), 1 (weak), 2 (moderate), or 3 (strong). An H-score was then derived by multiplying the staining intensity and the percentage of cells stained. Given that there is still no established cutoff value for CX3CL1 expression, a cutoff value of 10% immuno-positive tumor cells was recommended by the 3 experienced pathologists (**Figure S1**). Disagreement as to whether >10% of cells were positively stained was resolved by consensus.

### RNA Isolation and Quantitative PCR (qPCR)

Total RNA was extracted from tissues with TRIzol (Pufei, Shanghai, China) and retrotranscribed using PrimeScript RT Master Mix (Perfect Real Time; TaKaRa, Shiga, Japan) following the manufacturer’s recommendations. For qPCR, we used SYBR Premix Ex Taq (Tli RNaseH Plus; TaKaRa) and the 7500 Real-Time PCR System with Dell Tower (Applied Biosystems, Forster City, USA) system. The primers used to amplify CX3CL1, GAPDH, etc. were purchased from Ji Kai (Shanghai, China). The sequences of the primers for CX3CL1 were 5’-GTAGCTTTGCTCATCCACTATCA-3’ (upstream) and 5’-GACCACAGACTCGTCCATTCC-3’ (downstream). For each sample, the average value of the threshold cycle was normalized to the GAPDH level with the 2^-ΔΔCt^ method.

### Cell Lines and Viral Infection

T24 (RRID: CVCL_0554), 5637 (RRID: CVCL_0126) and TCCSUP (RRID: CVCL_1738) bladder urothelial cancer cells were purchased from Cell Bank of Chinese Academy of Sciences (Shanghai, China) and cultured in DMEM (Gibco, Waltham, USA) supplemented with 10% fetal bovine serum (FBS; PAA).

Approximately 1×10^6^ cells were cultured for 12 h to allow attachment to plates. Cells were rinsed once with PBS, and 10 mL of serum-free medium was added to the plate. We used an adenovirus (Ji Kai, Shanghai, China) that overexpressed shRNA targeting CX3CL1 to infect the cells at a multiplicity of infection (MOI) of 10. Sixteen hours after infection, the medium was replaced with 10 mL of complete medium.

### Cell Viability Assays and Transwell Assays

Cell viability was assessed at the indicated time points using an MTT kit (Gen-View, Calimesa, USA). MTT reagent (20 μL/well) was added to the medium, and the absorbance was read using a microplate reader at 490 nm for the test length and 570 nm for the reference. The percentage of viable cells was calculated based on the absorbance of the PBS control. Inserts in 24-well Transwell plates (Corning Costar, New York, USA) were coated with 500 μL/well Matrigel (BD Bioscience, San Jose, USA). Rehydration was performed with 500 μL/well serum-free medium for 2 h in a 37°C incubator (Sanyo, Japan). Samples were seeded into the inserts after removal of the rehydration medium. After 24 h of incubation, the cells that invaded through the membrane were stained with crystal violet (Beyotime, Shanghai, China) and observed at 200× magnification using a light microscope (Olympus, Tokyo, Japan).

### RNA-Seq Expression Profiling and Data Analysis

CX3CL1 was knocked down in TCCSUP cells by lentiviral infection [virus strain: LV-CX3CL1-RNAi (52620-1)]. RNA was extracted using the TRIzol method used for qPCR. cDNA was obtained by reverse transcription (Promega M-MLV kit) *via* a real-time qPCR detection system (Type Roche-LightCycler 480). The knockdown efficiency of the CX3CL1 gene was also evaluated by qPCR.

GeneChip PrimeView detection: RNA samples were subjected to quality inspection analysis (Agilent 2100 Bioanalyzer), and the GeneChip 3’IVT Express Kit was used to prepare amplified RNA (aRNA). That is, cDNA was obtained through first-strand synthesis, a double-stranded DNA template was obtained through second-strand synthesis, and then biotinylated aRNA was obtained by *in vitro* inversion. The aRNA was purified, fragmented, hybridized, washed with a chip probe (GeneChip Hybridization Wash and Stain Kit), and finally scanned to obtain images and raw data.

Statistical analysis and verification: Quality control was performed on the original data, statistical testing was used to identify differential genes between paired samples, and scatter plots, volcano plots, cluster plots and other chip analysis result plots were drawn. Ingenuity Pathway Analysis (IPA) database software was used to perform disease and function enrichment analysis and pathway enrichment analysis of the differentially expressed gene list; the results were verified by Western blot analysis.

### Xenografts and Animal Models

All experimental animals were obtained from Shanghai SLAC Laboratory Animal Co., Ltd. This work was carried out with 16 (8 male and 8 female) BALB/C nude mice aged 4-6 weeks and weighing 15 to 20 grams. All mice were kept under SPF living conditions. Mice were divided randomly and equally into 2 groups. The control group was named the negative control (NC) group and injected with normal TCCSUP cells, and the experimental group was named the KD group and injected with TCCSUP CX3CL1-knockdown cells. Each mouse was injected with 200 µl of cell suspension in the shoulder, which contained 1×10^7^ cells. We measured tumor volume every 5 days after injection, and calculated tumor volume with the following formula: Volume = 0.524 × L × W2 (L: long diameter; W: short diameter). The nude mice were sacrificed 35 days after injection, and tumor weight was measured. The subcutaneous tumors from each nude mouse were evaluated in pathological paraffin-embedded sections.

### Statistical Analysis

SPSS 21.0 (IBM, New York, USA) was used for statistical analyses. All data are presented as the mean ± standard deviation (SD). Student’s t-test and the Wilcoxon test were applied to compare gene expression and staining. Univariable and multivariable Cox/logistic regression analyses were used to evaluate the correlation between CX3CL1 expression and prognosis. Kaplan-Meier curve analyses and the log-rank test were also performed. A P value <0.05 was considered statistically significant.

## Results

The expression level of CX3CL1 mRNA in bladder cancer tissues was significantly higher than that in normal tissues (**Figure S2**, P=0.0268). A total of 277 patients were enrolled in the present study, with an average follow-up time of 49 months (1 to 72 months). Among them, 50 (18.1%) were negative for CX3CL1 expression, and 227 (81.9%) were considered positive for CX3CL1 expression. The demographic and clinical characteristics of the patients are summarized in **Table 1**. The patients were divided into 2 groups according to their expression of CX3CL1. By univariable analysis, the expression status of CX3CL1 was significantly associated with tumor stage [odds ratio (OR)=2.55, 95% confidence interval (95%CI): 1.36-4.76, P=0.003], tumor size (OR=2.06, 95%CI: 1.10-3.86, P=0.025), tumor grade (OR=1.91, 95%CI: 1.02-3.55, P=0.043), disease recurrence (OR=2.05, 95%CI: 1.08-3.88, P=0.029), metastatic disease (OR=3.98, 95%CI: 1.95-8.13, P<0.001), Ki67 expression (OR=1.02, 95%CI: 1.01-1.04, P=0.002) and mortality (OR=4.90, 95%CI: 2.53-9.47, P<0.001, **Table S1**). Multivariable analysis showed that CX3CL1 expression remained significantly associated with tumor size, tumor stage, Ki67 expression, disease recurrence, and mortality (all P<0.05).

**Table 1:** Clinical characteristics of entire cohort and subgroups by CX3CL1 expression.

Characteristics, n (%)	Entire Cohort (*N* = 270)	CX3CL1		P value
		Negative (*n* = 50)	Positive (*n* = 227)	
Age				
≤65 years	129 (46.6)	11 (8.5)	118 (91.5)	<0.0001
>65 years	148 (53.4)	39 (26.4)	109 (73.6)	
Gender				
Male	234 (84.5)	45 (19.2)	189 (80.8)	0.233
Female	43 (15.5)	5 (11.6)	38 (88.4)	
BMI				
<24	158 (57.0)	27 (17.1)	131 (82.9)	0.631
≥24	119 (43.0)	23 (19.3)	96 (80.7)	
Smoking				
No	194 (70.0)	32 (16.5)	162 (83.5)	0.262
Yes	83 (30.0)	18 (21.7)	65 (78.3)	
Tumor size				
<3cm	123 (44.4)	27 (22.0)	96 (78.0)	0.131
≥3cm	154 (55.6)	23 (14.9)	131 (85.1)	
No. of tumor sites				
Single	125 (45.1)	21 (16.8)	104 (83.2)	0.624
Multiple	152 (54.9)	29 (19.1)	123 (80.9)	
Tumor stage				
Ta-T1	188 (67.9)	25 (13.3)	163 (86.7)	0.003
T2-T4	89 (32.1)	25 (28.1)	64 (71.9)	
Tumor grade				
Low	147 (53.1)	20 (13.6)	127 (86.4)	0.041
High	130 (46.9)	30 (23.1)	100 (76.9)	
Ki67 expression				
<30%	171 (61.7)	34 (19.9)	137 (80.1)	0.314
≥30%	106 (38.3)	16 (15.1)	90 (84.9)	
Carcinoma *in situ*				
No	253 (91.3)	47 (18.6)	206 (81.4)	0.459
Yes	24 (8.7)	3 (12.5)	21 (87.5)	
Recurrence				
No	192 (69.3)	40 (20.8)	152 (79.2)	0.07
Yes	85 (30.7)	10 (11.8)	75 (88.2)	
Metastasis				
No	236 (85.2)	45 (19.1)	191 (80.9)	0.291
Yes	41 (14.8)	5 (12.2)	36 (87.8)	
Death				
No	226 (81.6)	46 (20.4)	180 (79.6)	0.036
Yes	51 (18.4)	4 (7.8)	47 (92.2)	

BMI, body mass index.

**Figure 1:**
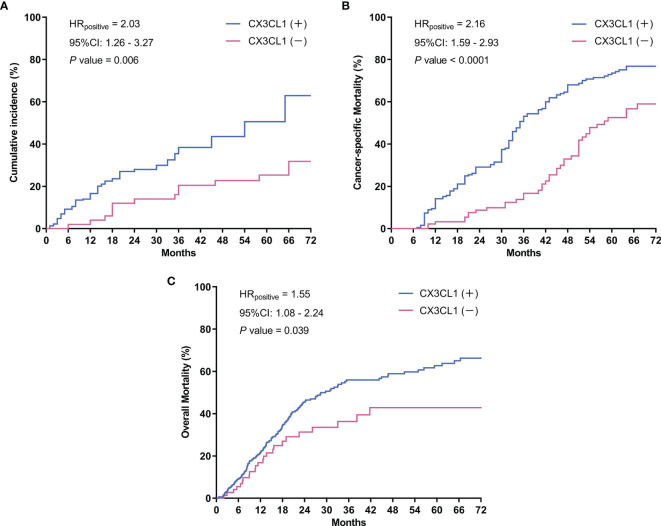
Survival curves. **(A)** Cumulative tumor recurrence rate: the blue line represents the cumulative recurrence rate of 277 patients with bladder cancer with positive CX3CL1 expression; the red line represents the CX3CL1 expression-negative group. **(B)** Cancer-specific survival: the blue line represents the cancer-specific survival of the bladder cancer CX3CL1-positive group; the red line represents the CX3CL1 expression-negative group. **(C)** Overall mortality in the TCGA database: the blue line represents the overall mortality associated with 406 bladder cancer samples obtained from the TCGA database with positive CX3CL1 expression; the red line represents the CX3CL1 expression-negative group. HR, hazard ratio; 95%CI, 95% confidence interval.

CX3CL1 was an independent predictor of BCa recurrence in both a univariable analysis (OR=2.44, 95%CI: 1.24-2.83, P=0.001) and a multivariable analysis (OR=2.37, 95%CI: 1.18-2.75, P=0.006) after adjusting for multiple variables, as shown in **Table 2**. As shown in the cumulative hazard curve of disease recurrence (**Figure 1A**), the 6-year cumulative recurrence rate of the BCa CX3CL1-positive group was 62.9%, which was significantly higher than that of the BCa CX3CL1-negative group (31.8%, P=0.006). Similarly, the BCa-specific survival rate of CX3CL1-positive group was 23.2%, which was significantly lower than that of the negative expression group (41.1%, P<0.0001, **Figure 1B**). Patients with high expression of CX3CL1 had an ~2-fold increased risk of cancer-specific death based on univariable (HR=2.16, 95%CI: 1.59-2.93, P<0.001) and multivariable (HR=2.17, 95%CI: 1.08-2.39, P=0.006) analyses (**Table 3**). This indicated that CX3CL1 was also a significant and independent risk factor for disease-specific death. A total of 406 BCa samples were obtained from the TCGA database. Survival analysis suggested that the patients with high expression of CX3CL1 (FPKM≥1.0) were significantly associated with poor survival compared with the patients with low CX3CL1 expression (log-rank P value=0.039, **Figure 1C**). These results remained significant after adjusting for age at diagnosis and tumor stage *via* Cox regression (HR=1.76, 95%CI: 1.14-2.71, P=0.011)

**Table 2:** Univariable and multivariable logistic regression analyses on tumor recurrence.

Predictors		Univariable analysis		Multivariable analysis	
		OR (95% CI)	*P* value	AOR (95% CI)	*P* value
Age	≤65 years	1.00 (Ref.)		/	/
	>65 years	1.11 (0.67-1.86)	0.679	/	/
Gender	Male	1.00 (Ref.)		/	/
	Female	1.35 (0.64-2.82)	0.431	/	/
BMI	<24	1.00 (Ref.)		1.00 (Ref.)	
	≥24	0.93 (0.86-1.00)	0.046	0.95 (0.88-1.03)	0.229
Smoking	No	1.00 (Ref.)		1.00 (Ref.)	
	Yes	1.80 (1.05-3.10)	0.033	1.98 (1.09-3.60)	0.025
Tumor size	<3cm	1.00 (Ref.)		1.00 (Ref.)	
	≥3cm	1.21 (1.03-1.42)	0.017	1.42 (1.05-1.91)	0.036
No. of tumor sites	Single	1.00 (Ref.)		/	/
	Multiple	1.45 (0.86-2.44)	0.162	/	/
Tumor stage	Ta-T1	1.00 (Ref.)		1.00 (Ref.)	
	T2-T4	2.41 (1.41-4.11)	0.001	1.83 (1.45-3.34)	0.027
Tumor grade	Low	1.00 (Ref.)		1.00 (Ref.)	
	High	2.30 (1.37-3.88)	0.002	1.82 (0.81-4.13)	0.011
Ki67 expression	<30%	1.00 (Ref.)		1.00 (Ref.)	
	≥30%	1.02 (1.01-1.04)	<0.001	1.02 (1.01-1.05)	0.002
Carcinoma *in situ*	No	1.00 (Ref.)		/	/
	Yes	0.92 (0.37-2.32)	0.866	/	/
CX3CL1	Negative	1.00 (Ref.)		1.00 (Ref.)	
	Positive	2.44 (1.24-2.83)	0.001	2.37 (1.18-2.75)	0.006

OR, odds ratio; 95% CI, 95% confidence interval; AOR, adjusted odds ratio; Ref, reference; BMI, body mass index.

**Table 3:** Univariable and multivariable-adjusted hazard ratios for cancer-specific mortality in Cox proportional hazards models.

Predictors		Univariable analysis		Multivariable analysis	
		HR (95% CI)	*P* value	AHR (95% CI)	*P* value
Age	≤65 years	1.00 (Ref.)		1.00 (Ref.)	
	>65 years	2.99 (1.59-5.62)	0.001	2.22 (1.08-4.57)	0.031
Gender	Male	1.00 (Ref.)		/	/
	Female	1.25 (0.55-2.86)	0.597	/	/
BMI	<24	1.00 (Ref.)		/	/
	≥24	0.95 (0.87-1.03)	0.223	/	/
Smoking	No	1.00 (Ref.)		/	/
	Yes	0.65 (0.34-1.27)	0.207	/	/
Tumor size	<3cm	1.00 (Ref.)		1.00 (Ref.)	
	≥3cm	2.19 (1.19-4.04)	0.012	2.26 (0.94-5.39)	0.067
No. of tumor sites	Single	1.00 (Ref.)		1.00 (Ref.)	
	Multiple	1.88 (1.03-3.41)	0.040	1.67 (1.29-3.51)	0.131
Tumor stage	Ta-T1	1.00 (Ref.)		1.00 (Ref.)	
	T2-T4	2.68 (1.49-4.82)	0.001	1.77 (1.30-3.96)	0.178
Tumor grade	Low	1.00 (Ref.)		1.00 (Ref.)	
	High	3.42 (1.85-6.33)	<0.001	2.59 (1.06-6.31)	0.037
Ki67 expression	<30%	1.00 (Ref.)		1.00 (Ref.)	
	≥30%	1.02 (1.01-1.03)	0.001	1.03 (1.01-1.04)	0.002
Carcinoma *in situ*	No	1.00 (Ref.)		/	/
	Yes	0.81 (0.77-1.34)	0.116	/	/
CX3CL1	Negative	1.00 (Ref.)		1.00 (Ref.)	
	Positive	2.16 (1.59-2.93)	<0.001	2.17 (1.08-2.39)	0.006

HR, hazard ratio; 95% CI, 95% confidence interval; AHR, adjusted hazard ratio; Ref, reference; BMI, body mass index.

**Figure 2:**
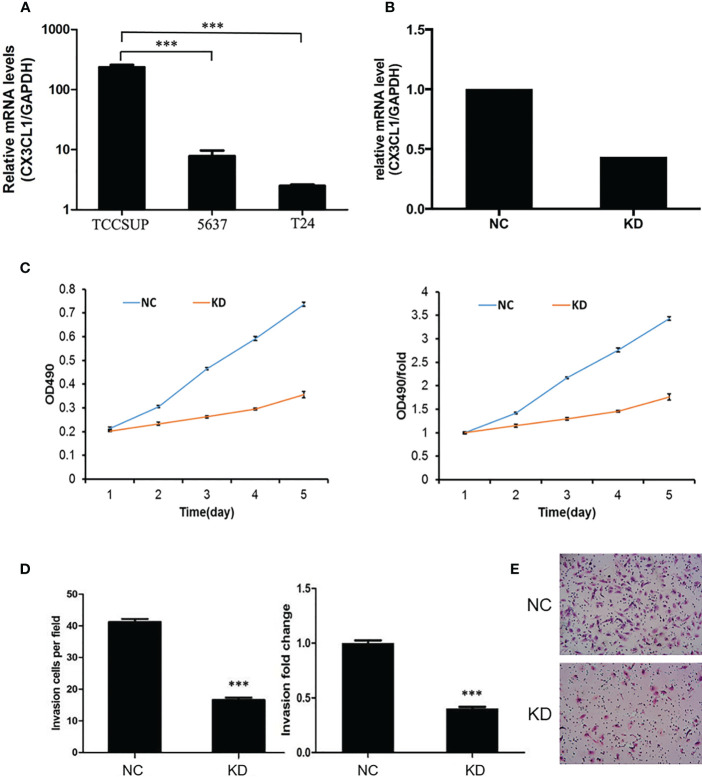
**(A)** The mRNA expression of CX3CL1 in TCCSUP, 5637 and T24 cells. The mRNA expression of CX3CL1 in TCCSUP cells was significantly higher than that in 5637 and T24 cells. **(B)** Difference in CX3CL1 mRNA levels between NC and KD TCCSUP cells. The CX3CL1 gene of TCCSUP cells in KD group was knocked down, and the knockdown rate was verified by qPCR. **(C)** Difference in TCCSUP cell proliferation between NC and KD groups determined by the MTT method. The results showed that cell proliferation in the KD group(Orange line) was significantly decreased. **(D, E)** Cell invasion of NC and KD cells determined by Transwell assays. The results showed that after CX3CL1 was knocked down, cell invasion in the KD group was significantly reduced. ***P < 0.001.

To evaluate whether CX3CL1 expression is associated with disease aggressiveness with functional experiments, we evaluated the expression levels in 3 different types of cell lines, TCCSUP (grade IV cancer), 5637 (grade II cancer), and T24 cells (high-grade and invasive TCC). The mRNA expression of CX3CL1 in TCCSUP cells was significantly higher than that in 5637 and T24 cells (P<0.001, **Figure 2A**). No significant difference in CX3CL1 expression was observed between the 5637 and T24 cell lines (P=0.766). Thus, TCCSUP cells were used for further evaluation. CX3CL1 was then knocked down by lentiviral infection. The knockdown efficiency was then verified by qPCR (efficiency reached 56.5%, P=0.0032, **Figure 2B**). We then evaluated whether CX3CL1 expression is associated with cancer cell proliferation and invasiveness. MTT assay results showed that TCCSUP cell proliferation was significantly decreased in the KD group compared to the NC group (**Figure 2C**, P<0.001). Transwell assays suggested a significant decrease in cell invasion in the KD group compared with the NC group (**Figures 2D, E**, P<0.001).

**Figure 3:**
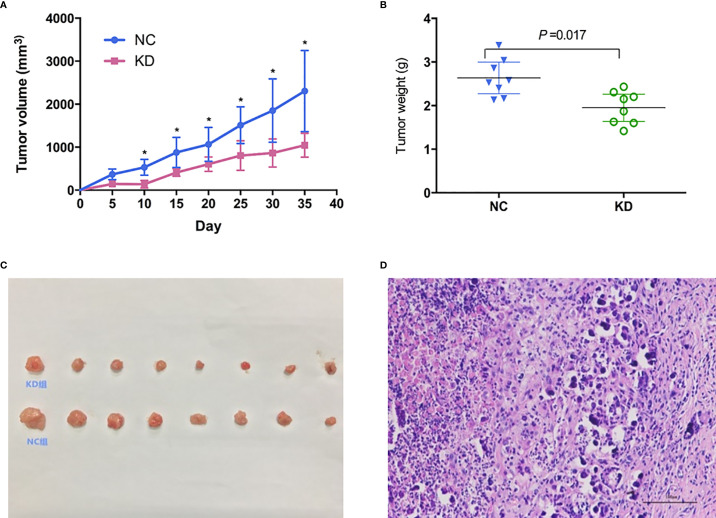
Effects of CX3CL1 knockdown on **(A)** tumor volume and **(B)** weight changes in a nude mouse subcutaneous tumor model of bladder cancer. The results showed that compared with the control group, the volume and weight of subcutaneous tumors in the KD group decreased significantly. **(C)** Subcutaneous tumor in KD (up) and NC (down) group. **(D)** Hematoxylin & Eosin staining of the tumor cells. KD, knockdown; NC, negative control.

To further evaluate whether CX3CL1 expression is associated with BCa aggressiveness *in vivo*, we established xenografts in the shoulder of immunocompromised nude mice. Subcutaneous tumor formation in the nude mice was verified by pathologists, and the success rate was 100% (**Figure 3**). From the 10th day to the 35th day, the tumor volume in the KD group was significantly smaller than that in the NC group (**Figure 3**, days 10-35, P<0.05). The tumor weight in the KD group was significantly lower than that in the NC group (**Figure 3**, day 35, P=0.017).

**Figure 4:**
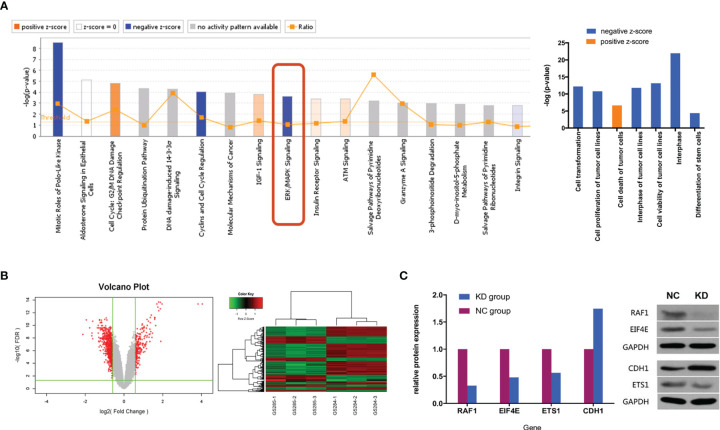
**(A)** Classic pathway enrichment analysis and disease and function analysis based on IPA by GeneChip PrimeView. Our experiments suggested that when CX3CL1 is inhibited, the ERK/MAPK signaling pathway is inhibited, the function of promoting tumor growth is inhibited, and tumor death is enhanced. **(B)** Chip volcano plot (left) and hierarchical clustering (right): in the volcano plot, the abscissa represents the multiple of the gene expression difference (transformed by log2), the ordinate represents the significant FDR of the difference (transformed by log10), the red dots were selected with |Fold Change|>1.5 and FDR<0.05 as the screening criteria for genes that were significantly differentially expressed, and the gray dots are other genes that were not significantly differentially expressed; in the hierarchical clustering data, red means that gene expression was relatively upregulated, green means that gene expression was relatively downregulated, black means that the degree of gene expression did not change significantly, and gray means that the signal intensity of the gene was not detected. **(C)** Relative expression of downstream genes and proteins in the ERK/MAPK signaling pathway between NC and KD cells (Western blot). NC, normal control group; KD, CX3CL1 gene knockdown group.

To investigate potential biological functions, we performed gene expression profiling using an mRNA microarray. Totals of 182 and 367 genes were found to be significantly upregulated and downregulated, respectively, in CX3CL1 KD TCCSUP cells. IPA-based disease and function analysis suggested that after CX3CL1 was inhibited, tumor cell proliferation (P=1.62×10^-11^, z-score=-2.32), cell malignant transformation (P=6.47×10^-13^, z-score=-2.289), tumor cell interphase (P=1.57×10^-12^, z-score=-2.078) and differentiation of stem cells (P=4.02×10^-5^, z-score=-2.465) were significantly inhibited, and tumor cell apoptosis (P=2.35×10^-7^, z-score=2.236) was significantly increased (**Figure 4A**). CDH1, ETS1, RAF1, and EIF4E were genes with major effects in the enrichment analysis. In terms of classic pathway enrichment analysis based on IPA, the ERK/MAPK signaling pathway was significantly inhibited (P=2.19×10^-4^, z-score=-1.604). ETS1, RAF1, and EIF4E were closely related to the ERK/MAPK pathway and significantly downregulated (**Figure 4A** and **Table S2**). A volcano plot and hierarchical clustering for significant difference analysis are shown in **Figure 4B**. Briefly, the tumor cell apoptosis (P=2.35×10^-7^, z-score=2.236) pathway was significantly activated, while tumor cell proliferation (P=1.62×10^-11^, z-score=-2.32), tumor cell interphase (P=1.57×10^-12^, z-score=-2.078), cell viability of tumor cell lines (P=7.12×10^-14^, z-score=-2.092), differentiation of stem cells (P=4.02×10^-5^, z-score=-2.465), cell malignant transformation (P=6.47×10^-13^, z-score=-2.289), etc. were significantly inhibited.

To further confirm whether the genes in the ERK/MAPK pathway are influenced by the expression of CX3CL1, the protein expression of the enriched genes including RAF1, EIF4E, ETS1, and CDH1 was evaluated by Western blotting. The results suggested that RAF1, EIF4E, and ETS1 protein expression was significantly downregulated by 67.2%, 52.0%, and 43.6%, respectively (all P<0.05), while CDH1 protein expression was significantly upregulated by 74.6% (P<0.05, **Figure 4C**).

## Discussion

Tumor immunotherapy is considered as an important and hopeful curative therapy for cancers, such as PD-1/PD-L1. Immune surveillance and immune tolerance usually serve with a two-side effect throughout cancer development and progression. Scientists have found that chemokines family was the key factor in this process by affecting the immune system (3). Among different chemokines, CX3CL1 is considered as one of the most important one, and may enhance local anti-tumor immunity by polarizing immunocompetent cells around the tumor microenvironment, and therefore inhibit tumor growth (4); on the other hand, tumors spontaneously secrete chemokines and receptors, which can promote the growth of cancer cells (5). We found that high expression of CX3CL1 was significantly associated with higher risk of disease recurrence and cancer-specific death; through *in vivo* and *in vitro* functional study, we further confirmed that high expression of CX3CL1 was associated with tumor cell proliferation and invasion. Enrichment analysis and functional study based on expression array data suggested that the regulation of tumor growth by CX3CL1 might due to the enhance of the ERK/MAPK signaling pathway.

CX3CL1 is a large cytokine (373 amino acids) and the only known member of the CX3C chemokine family. It is also known as fractalkine (in humans) and neurotactin (in mice) (6, 20). It has been proved correlating with cell migration and interaction in multiple diseases. Shulby *et al.* presented the first evidence of the expression of CX3CR1 (the specific receptor of fractalkine) in human prostate cancer cells and the expression of fractalkine in human bone marrow endothelial cells. They also found that a neutralizing antibody against fractalkine might significantly reduce the adhesion of prostate cancer cells to human bone marrow endothelial cells, suggesting the important role of fractalkine in this process (13). By silencing CX3CR1, Jamieson et al. further defined the roles of this chemokine receptor in the adhesion of prostate cancer cells to bone marrow endothelial cells and prostate cancer cell migration towards media conditioned by different types of bone cells (21). Tardaguila et al. found that CX3CL1 expression was downregulated in HER2/neu tumors; however, paradoxically, adenovirus-mediated CX3CL1 expression in the tumor milieu enhanced mammary tumor numbers in a dose-dependent manner. Additionally, CX3CL1 triggered cell proliferation by inducing ErbB receptors through the proteolytic shedding of an ErbB ligand, and these findings support the conclusion that CX3CL1 acts as a positive modifier of breast cancer in concert with ErbB receptors (22). In another study on breast cancer, Tsang et al. reported that high CX3CL1 expression was detected in 33.3% of primary invasive cancers and that CX3CL1 expression correlated positively with increased tumor-infiltrating lymphocytes (TILs; P=0.005). Furthermore, adverse features in breast cancers, including lymph node involvement, α-β crystalline expression and high Ki67, were positively correlated with the expression of CX3CL1. Notably, in the same study, the authors showed that patients with high levels of CX3CL1 had poorer overall survival. The findings indicated an oncogenic role for CX3CL1, despite its previously suggested role in enhancing antitumor immunity, and highlighted the complicated functions of CX3CL1 in breast carcinogenesis (23). However, a study on the prognosis of patients with liver cancer showed that patients with high expression of CX3CL1 and its receptor had a better prognosis than those with low expression, with a significantly lower local recurrence rate and distant metastasis rate and longer disease-free survival and overall survival (15). These results indicate that CX3CL1 has complex functions in tumors. At present, there are few in-depth studies on the role of CX3CL1 in BCa, and the mechanism of action of the chemokine CX3CL1 is still unclear. We conducted a series of experimental studies to investigate the role of CX3CL1 in BCa.

Previous studies have shown that CX3CL1 may promote or suppress tumors in different tissues. Our research confirms the value and function of CX3CL1 in BCa. Compared with previous studies, our study identified possible signaling pathways and downstream genes of CX3CL1 in BCa through GeneChip PrimeView Human technology and explored the possible mechanisms in more depth. Through bioinformatic data analysis and statistics, the significant differences were further verified. We found that after CX3CL1 was inhibited, tumor cell proliferation, cell malignant transformation, tumor cell interphase, and differentiation of stem cells were significantly inhibited, and tumor cell apoptosis was significantly increased. These results suggest that CX3CL1 may play a role in promoting the proliferation of BCa cells. In this experiment, the ERK/MAPK pathway was significantly inhibited, and the downstream genes ETS1 (↓), RAF1 (↓), and EIF4E (↓), which are closely related to this pathway, were significantly downregulated. In addition, the downstream gene CDH1 (↑) was significantly upregulated. Further detection of the expression of the proteins encoded by the above genes was also confirmed. The RNA-Seq results, and protein expression results are consistent with a previous clinical prognostic analysis and *in vivo* and *in vitro* experimental results, which together indicate the tumor-promotive effect of CX3CL1 in BCa.

This study suggests that CX3CL1 might promote the cancer progression through the ERK/MAPK signaling pathway. Mitogen-activated protein kinase (MAPK) is a threonine/serine protein kinase that is widely present in cells. It is closely related to cell proliferation, apoptosis, tumor proliferation, invasion and metastasis. The MAPK pathway is one of the most studied signaling pathways in recent years (24, 25). Among MAPK family members, extracellular signal-regulated kinase (ERK) was the first discovered and is the most widely studied member. An abnormal ERK/MAPK pathway can lead to abnormal cell proliferation, which is related to tumorigenesis and can further affect the biological behavior of tumors (26). Current studies indicate that the ERK/MAPK signaling pathway plays an important role in tumor occurrence and development by promoting cancer cell proliferation, inhibiting tumor apoptosis, and inducing tumor neovascularization, invasion, and metastasis (27–30). Studies have also pointed out that the ERK/MAPK signaling pathway is also related to tumor resistance (31, 32). To date, many studies have found that the ERK/MAPK signaling pathway is related to many tumors, such as colon cancer, gastric cancer, liver cancer, melanoma, and BCa (33–38). According to previous research reports, among the four significantly regulated downstream genes found in this experiment, CDH1 was considered to be an anti-oncogene, and ETS1, RAF1 and EIF4E play roles in promoting cancer (39–48). Our research results are consistent with those in previous research reports.

To the best of our knowledge, this was the first study investigating the association between CX3CL1 expression and BCa. The results showed that CX3CL1 overexpression was independently and significantly associated with poor prognosis (increased risk of recurrence and cancer-specific death rate). In addition, we were able to confirm our results in TCGA dataset. This finding may be applied to clinical practice as a valuable prognostic biomarker *via* additional translational research approaches.

Our study has carried out a series of elaboration on the role of CX3CL1 in BCa. However, several limitations should be noted in the present study. First, the sample size was relatively small. However, we were able to validate our results in TCGA database. The results should be investigated in a large-scale study in the future. Second, the subcutaneous xenograft animal model could not fully simulate the tumor microenvironment. Although orthotopic bladder cancer model would be a better option, the current method was the most common used model in BCa research. Last but not least, only one cell line was chosen in the present study. However, the main objective of this study was to evaluate the association of CX3CL1 expression and BCa prognosis.

## Conclusion

CX3CL1 was a significant and independent predictor for bladder cancer prognosis. It could promote the proliferation and invasion of bladder cancer cells *via* inhibiting the ERK/MAPK signaling pathway.

## Data Availability Statement

The raw data supporting the conclusions of this article will be made available by the authors, without undue reservation.

## Ethics Statement

The studies involving human participants were reviewed and approved by The Institutional Review Board at Huashan Hospital. The written informed consent was obtained from all patients for institutional biobank. The animal study was reviewed and approved by The Institutional Review Board at Huashan Hospital.

## Author Contributions

KX, RN, and NZ conceived and designed the study. GJ, HW, YW, WD, and QZ acquired the data. GJ, HW, and DH analyzed and interpreted the data. GJ, HW, and DH drafted the manuscript. KX, RN, NZ, and QD contributed to the critical revision of the manuscript. KX and RN supervised the study. All authors contributed to the article and approved the submitted version.

## Funding

This research was supported by the National Natural Science Foundation of China (Grant No. 81472379 and 81402339).

## Conflict of Interest

The authors declare that the research was conducted in the absence of any commercial or financial relationships that could be construed as a potential conflict of interest.

The reviewer HW declared a shared affiliation, with no collaboration, with several of the authors HW, YW, WD, QZ, QD, KX to the handling editor at the time of review.

## Publisher’s Note

All claims expressed in this article are solely those of the authors and do not necessarily represent those of their affiliated organizations, or those of the publisher, the editors and the reviewers. Any product that may be evaluated in this article, or claim that may be made by its manufacturer, is not guaranteed or endorsed by the publisher.
