# Supplementary material for: The Clinical Implications and Molecular Mechanism of CX3CL1 Expression in Urothelial Bladder Cancer
Source: Front Oncol. 2021 Oct 4;11:752860. doi: 10.3389/fonc.2021.752860 (PMC8521074; doi:10.3389/fonc.2021.752860)
Supplement: Supplementary file 1 [file DataSheet_1.docx]

Supplementary Material

**Supplementary Figure S1** CX3CL1 positive or negative in bladder cancer slices


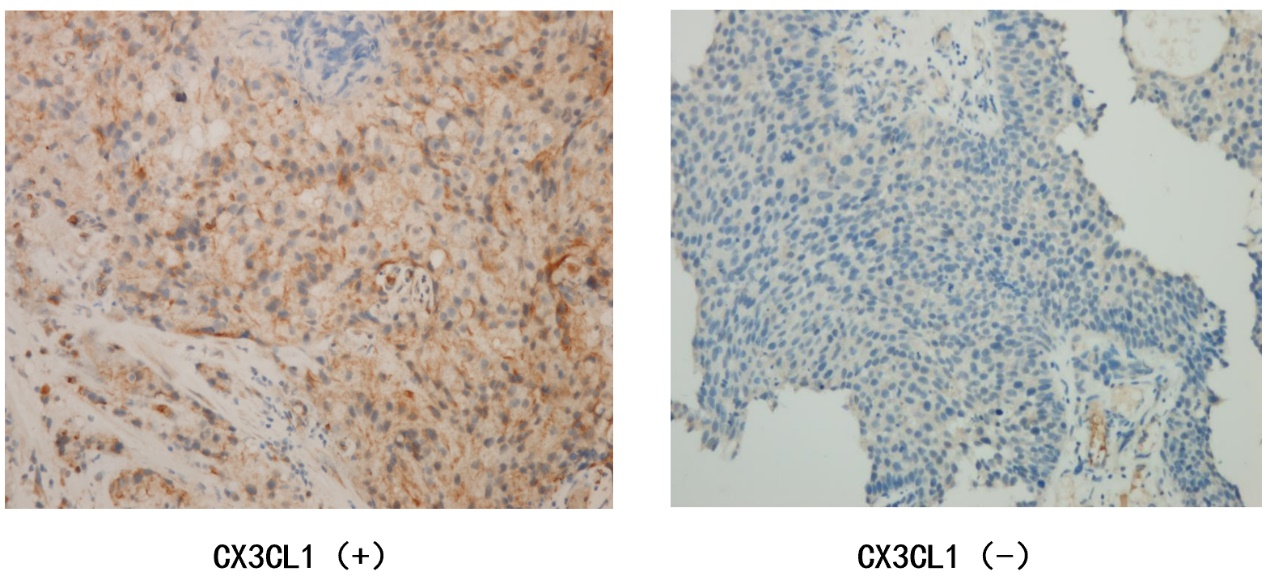


**Supplementary Figure S2** The relative value of CX3CL1 mRNA levels in Real-Time PCR (4 cases in normal tissue group; 5 cases in tumor tissue group).


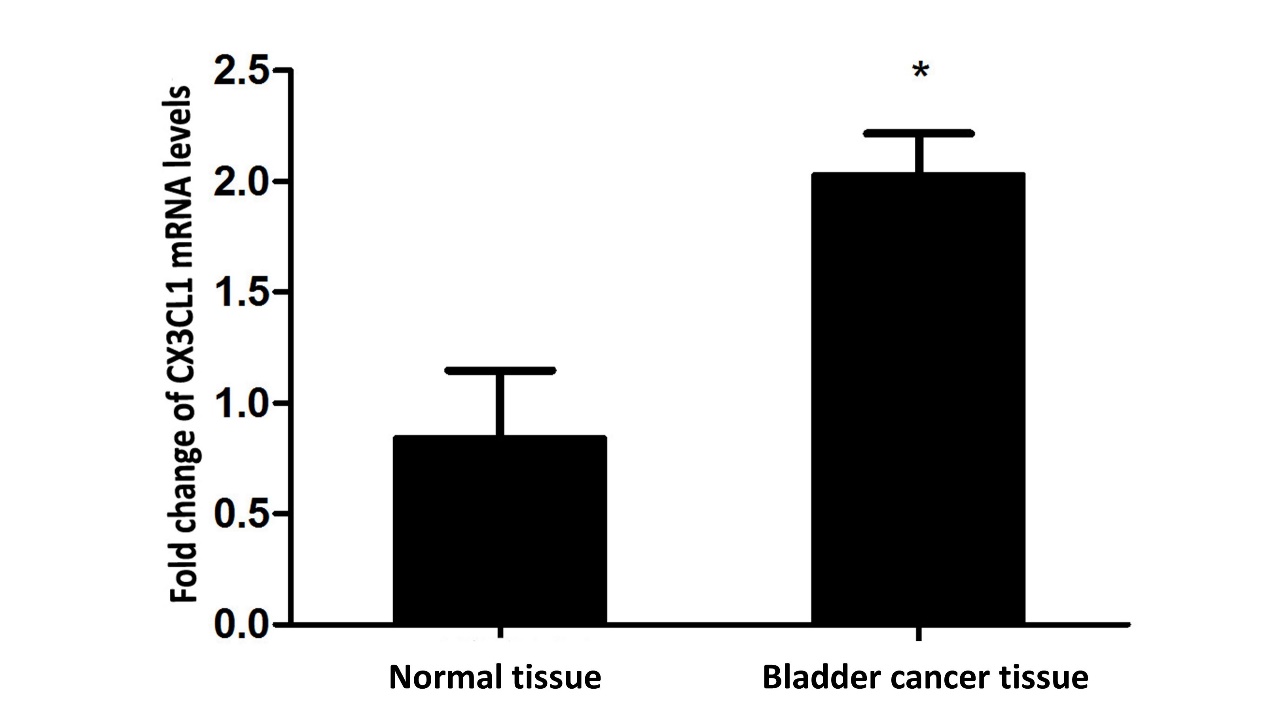


**Table S1** Univariable and multivariable logistic regression analyses on CX3CL1 expression (positive versus negative [reference]).

| Characteristics | | Univariable analysis | |  | Multivariable analysis | |
| --- | --- | --- | --- | --- | --- | --- |
|  |  | OR (95% CI) | *P* value |  | AOR (95% CI) | *P* value |
| Age | ≤65 years | 1.00 (Ref.) |  |  | 1.00 (Ref.) |  |
|  | >65 years | 3.84 (1.87-7.87) | <0.001 |  | 2.74 (1.13-6.64) | 0.026 |
| Gender | Male | 1.00 (Ref.) |  |  |  |  |
|  | Female | 1.81 (0.67-4.86) | 0.239 |  |  |  |
| BMI | <24 | 1.00 (Ref.) |  |  |  |  |
|  | ≥24 | 1.03 (0.94-1.12) | 0.512 |  |  |  |
| Smoking | No | 1.00 (Ref.) |  |  |  |  |
|  | Yes | 1.39 (0.73-2.64) | 0.323 |  |  |  |
| Tumor size | <3cm | 1.00 (Ref.) |  |  | 1.00 (Ref.) |  |
|  | ≥3cm | 2.06 (1.10-3.86) | 0.025 |  | 7.85 (1.78-9.75) | 0.007 |
| No. of tumor sites | Single | 1.00 (Ref.) |  |  |  |  |
|  | Multiple | 1.17 (0.63-2.17) | 0.624 |  |  |  |
| Tumor stage | Ta-T1 | 1.00 (Ref.) |  |  | 1.00 (Ref.) |  |
|  | T2-T4 | 2.55 (1.36-4.76) | 0.003 |  | 6.10 (2.84-9.88) | <0.001 |
| Tumor grade | Low | 1.00 (Ref.) |  |  | 1.00 (Ref.) |  |
|  | High | 1.91 (1.02-3.55) | 0.043 |  | 1.99 (0.71-5.60) | 0.192 |
| Ki67 expression | <30% | 1.00 (Ref.) |  |  | 1.00 (Ref.) |  |
|  | ≥30% | 1.02 (1.01-1.04) | 0.002 |  | 0.96 (0.93-0.98) | 0.001 |
| Carcinoma in situ | No | 1.00 (Ref.) |  |  |  |  |
|  | Yes | 1.60 (0.46-5.58) | 0.463 |  |  |  |
| Recurrence | No | 1.00 (Ref.) |  |  | 1.00 (Ref.) |  |
|  | Yes | 2.05 (1.08-3.88) | 0.029 |  | 2.34 (1.15-3.78) | 0.011 |
| Metastasis | No | 1.00 (Ref.) |  |  | 1.00 (Ref.) |  |
|  | Yes | 3.98 (1.95-8.13) | <0.001 |  | 1.43 (0.34-6.10) | 0.628 |
| Death | No | 1.00 (Ref.) |  |  | 1.00 (Ref.) |  |
|  | Yes | 4.90 (2.53-9.47) | <0.001 |  | 4.73 (1.26-7.76) | 0.021 |

Abbreviation: OR, odds ratio; 95% CI, 95% confidence interval; AOR, adjusted odds ratio; Ref, reference; BMI, body mass index.

**Table S2** Disease and function enrichment analysis

| Diseases or Functions Annotation | *P* value | Predicted  Activation State | Activation z-score | Molecules |  |
| --- | --- | --- | --- | --- | --- |
|  |  |  |  |  |  |
| Cell transformation | 6.47E-13 | Decreased | -2.289 | AFDN, CDH1, CHKA, EIF4E, ETS1, FOS, RAF1, RBL2, YAP1, *et al*. |  |
| Cell proliferation of tumor cell lines | 1.62E-11 | Decreased | -2.32 | BEX2, CDH1, CDK1, EIF4E, ETS1, RAF1, STK38, WSB1, *et al.* |  |
| Cell death of tumor cells | 2.35E-07 | Increased | 2.236 | CDH1, ETS1, FOS, HBEGF, RAF1, *et al.* |  |
| Interphase of tumor cell lines | 1.57E-12 | Decreased | -2.078 | CDH1, DESI2, ETS1, RAF1, WEE1, *et al.* |  |
| Cell viability of tumor cell lines | 7.12E-14 | Decreased | -2.092 | CCNB1, CDH1, EIF4E, NUF2, RAF1, *et al.* |  |
| Interphase | 1.21E-22 | Decreased | -2.153 | CDH1, EIF4E, ETS1, HMOX1, ID3, RAF1, *et al.* |  |
| Differentiation of stem cells | 4.02E-05 | Decreased | -2.465 | CDH1,PIM1, RBL2, YAP1, *et al.* |  |
